# Adiposity is Associated with Decreased Serum 17-Hydroxyprogesterone Levels in Non-Diabetic Obese Men Aged 18–49: A Cross-Sectional Study

**DOI:** 10.3390/jcm9123873

**Published:** 2020-11-28

**Authors:** José Ignacio Martínez-Montoro, María Molina-Vega, Maite Asenjo-Plaza, María Concepción García-Ruiz, Enrique Varea-Marineto, Isaac Plaza-Andrade, Juan J. Álvarez-Millán, Pablo Cabezas-Sánchez, Francisco J. Tinahones, José Carlos Fernández-García

**Affiliations:** 1 Department of Endocrinology and Nutrition, Virgen de la Victoria University Hospital, 29010 Málaga, Spain; joseimartinezmontoro@gmail.com (J.I.M.-M.); josecarlosfdezgarcia@hotmail.com (J.C.F.-G.); 2 Laboratorio de Investigación, Instituto de Investigación Biomédica de Málaga (IBIMA), 29010 Málaga, Spain; isaacplazaandrade@gmail.com; 3 Cruz de Humilladero Primary Care Centre, 29006 Málaga, Spain; maite.asenjo.plaza@gmail.com (M.A.-P.); adagaru60@gmail.com (M.C.G.-R.); envarneto@hotmail.com (E.V.-M.); 4 Consulting Químico Sanitario (CQS Lab), 28521 Madrid, Spain; jamillan@cqslab.com (J.J.Á.-M.); pcabezas@cqslab.com (P.C.-S.); 5 Centro de Investigación Biomédica en Red de la Fisiopatología de la Obesidad y Nutrición (CIBEROBN), Instituto de Salud Carlos III (ISCIII), 28029 Madrid, Spain; 6 Endocrinology and Nutrition Department, Regional University Hospital of Malaga, 29010 Malaga, Spain

**Keywords:** obesity, visceral fat, 17-hydroxyprogesterone, hypogonadism

## Abstract

Obesity is associated with decreased circulating testosterone levels, the main male sex hormone. However, there are a number of different male sex hormones whose dynamics remain poorly understood regarding this pathology. In this regard, 17 hydroxyprogesterone (17-OH progesterone), as an important precursor of testosterone synthetized in testes and adrenal glands, could play an essential role in testosterone deficiency in male obesity. Moreover, similarly to testosterone, 17-OH progesterone could be closely associated with visceral fat distribution and metabolic dysfunction. Thus, the aim of this study was to assess serum 17-OH progesterone levels in non-diabetic obese young men and to evaluate their relationship with clinical, analytical, and anthropometric parameters. We conducted a cross-sectional study including 266 non-diabetic men with obesity (BMI ≥ 30 kg/m^2^) aged 18–49 years; 17-OH progesterone and total testosterone (TT) were determined by high-performance liquid chromatography mass spectrometry. 17-OH progesterone levels were significantly lower in tertile 3 of body fat percentage in comparison with tertile 1 (0.74 ng/mL vs. 0.94 ng/mL, *p* < 0.01; Bonferroni correction) and in comparison with tertile 2 (0.74 ng/mL vs. 0.89 ng/mL, *p* = 0.02; Bonferroni correction). 17-OH progesterone levels correlated negatively with weight, BMI, waist circumference, insulin, homeostatic model assessment of insulin resistance (HOMA-IR), and visceral fat, and positively with TT, free testosterone (FT), luteinizing hormone, and fat-free mass percentage. Multivariate linear-regression analysis showed that body fat percentage and HOMA-IR were inversely associated with 17-OH progesterone levels, while FT and ACTH were positively linked to circulating 17-OH progesterone levels. In conclusion, in a population of non-diabetic obese young men, 17-OH progesterone levels were inversely associated with adiposity. Body fat percentage and insulin resistance were negatively related to 17-OH progesterone levels, whereas FT and ACTH levels were positively associated with 17-OH progesterone levels.

## 1. Introduction

Overweight and obesity worldwide prevalence has increased in pandemic dimensions in the last few decades. Globally, the estimated number of overweight and obese adults in 2015 was 1.9 billion and 609 million, respectively, which represents 39% of the world’s population [1]. Obesity is associated with a major number of comorbidities, such as type 2 diabetes, cardiovascular disease, cancer, or obstructive sleep apnea [2]. It is also considered the most frequent cause of male hypogonadism [3]. The prevalence of male hypogonadism has been reported to be more than 40% in obese males [4]. Although some conditions usually associated with obesity, such as type 2 diabetes or cardiovascular disease, are closely linked to testosterone decrease, even in young obese men without these comorbidities, the prevalence of hypogonadism remains high, affecting more than 25% of these subjects. Moreover, the hypogonadism prevalence rises >75% in males with extreme obesity [5].

Androgens play a major role in sexual development and function. Although testosterone is the main circulating male hormone, there are other testicular and adrenal androgenic steroids whose role remains uncertain. In this line, 17-hydroxyprogesterone (17-OH progesterone) is a C_21_ steroid derived from progesterone via 17-hydroxylase or 17-hydroxypregnenolone via 3β-hydroxysteroid dehydrogenase/Δ5-4 isomerase, that acts as intermediate in the biosynthesis of hydrocortisone and gonadal steroid hormones [6]. In men, most serum 17-OH progesterone has a testicular and not adrenal origin; approximately 70% of 17-OH progesterone is synthetized in testes, while the remaining production comes from adrenal glands. Similar to testosterone, 17-OH progesterone is stimulated by the luteinizing hormone (LH) and the human chorionic gonadotropin (hCG) [7,8,9]. Decreased serum testosterone levels in obesity are linked to reduced C_19_ steroids biosynthesis and potentially, this reduction could carry an altered metabolic risk profile [10,11].

Accordingly, a compromised synthesis of C_21_ steroid 17-OH progesterone could constitute a previous step in the impaired C_19_ steroids and testosterone production in obese men. However, the lack of knowledge about the specific role of 17-OH progesterone has motivated several hypotheses, mainly connected to spermatogenesis and fertility. Moreover, as 17-OH progesterone is the second-most important intratesticular steroid in percentage after testosterone, and given the variability between serum testosterone levels and intratesticular testosterone levels, some authors have proposed that circulating 17-OH progesterone could be a more useful serum marker for intratesticular testosterone concentrations and might be helpful to monitor patients receiving hormonal treatment for male infertility and to titrate the dose of medication [12,13]. 

In contrast to the well-documented relationship between obesity and serum testosterone levels, there is limited knowledge about 17-OH progesterone dynamics in obese subjects. In this regard, previous studies have reported decreased circulating levels of different androgens and their precursors in obese men, including 17-OH progesterone, although none of them were specifically focused on this steroid hormone and they only measured this parameter in a reduced number of participants [14,15,16]. Moreover, the presence of type 2 diabetes, cardiovascular disease, or aging could constitute confounding variables in the evaluation of 17-OH progesterone. Furthermore, the assessment of body composition analysis in these patients could shed light about the relationship between 17-OH-progesterone and adiposity. 

Hence, in the present work we aimed to assess the dynamics of 17-OH progesterone in nondiabetic obese young men and to evaluate if their circulating levels are decreased in parallel to adiposity. Also, we evaluated the relationship between 17-OH progesterone concentrations with clinical, analytical, and anthropometric parameters. 

## 2. Experimental Section

### 2.1. Study Design and Participants

This was a cross-sectional study, done at the Virgen de la Victoria University Hospital (Malaga, Spain), from June 2013 to June 2015. Men with obesity (BMI ≥ 30 kg/m^2^) aged 18–49 years old were included in this study. Those men with a previous diagnosis of hypoandrogenemia, diabetes mellitus (diagnosed if a potential participant was taking medication for diabetes, had fasting plasma glucose ≥126 mg per deciliter (7 mmol per liter)), or HbA1c ≥6.5%, as confirmed by repeated testing), use of any antidiabetic medication, or being under any treatment known to affect the gonadal axis, including any form of testosterone were excluded. The existence of hepatic impairment (total bilirubin levels > 2 mg/dL or aspartate amino transferase levels three times higher than the normal upper limit) or renal impairment (estimated glomerular filtration rate < 60 mL/min/1.73 m^2^ or albumin to creatinine ratio ≥ 30 mg/g), established cardiovascular disease (defined as documented ischemic heart disease, cerebrovascular disease, or peripheral arterial disease) or previous history of cancer (except basalioma), or active cancer of any kind was also exclusion criteria. 

### 2.2. Biochemical Evaluation

Blood samples were collected from all participants between 08:00 and 10:00 after 10 h of fasting. Study participants were instructed to eat a light meal the evening before. Samples were centrifuged and plasma and serum were distributed in aliquots and stored at −80 °C until analysis.

In order to obtain age, medical history, and current diseases and associated treatment, a structured interview was completed by participants. We also collected weight and height (to calculate BMI) and waist circumference (WC). Biochemical parameters were measured in duplicate using standard enzymatic methods. Homeostatic model assessment of insulin resistance (HOMA-IR), as described by Mathews et al. [17] was used to determine insulin resistance. High-sensitivity C-reactive protein (hs-CRP) was analyzed in a multiplex immunoassay platform, Adrenocorticotropic hormone (ACTH) was measured in EDTA (ethylenediaminetetraacetic acid) plasma using a two-site sequential chemiluminescent immunometric assay (reference values 5–50 pg/mL). Luteinizing hormone (LH) was determined using a direct quimiluminometric assay (reference values 1.5–7.7 mIU/mL). Total testosterone (TT) was determined by high-performance liquid chromatography mass spectrometry (HPLC-MS), details about its determination can be found elsewhere [5]. Sex-hormone-binding globulin was determined with an electrochemiluminescence immunoassay (reference range 15–50 nmol/L) and free testosterone (FT) was calculated from TT and sex-hormone-binding globulin using a law-of-mass-action equation [18]. 17-OH progesterone was determined by HPLC-MS conducted in a triple quadrupole liquid chromatography mass spectrometry system (Model 6460; Agilent Technologies, Santa Clara, California). The lower limit of detection was 0.062 ng/mL, the interassay coefficient of variation was 2.66% at 0.30 ng/mL, 3.89% at 1.46 ng/mL, and 0.76% at 8.97 ng/mL, and the intraassay coefficient of variation was 2.18% at 0.30 ng/mL, 4.19% at 1.46 ng/mL, and 1.77% at 8.97 ng/mL. Accuracy was 102%, and recovery was 92%. The calibrators were human serum samples. The material was lyophilized and contained six levels of 0.097, 0.48, 0.961, 1.95, 3.85, and 21.6 ug/L and a blank. Quality control (QC) samples were prepared from different stock solution at three levels of human serum samples (0.3, 1.46, and 8.970 ug/L serum concentration). Working calibrations and QC were reconstituted with 3 mL of distilled water into each vial and incubated 15 min at room temperature. The vials were swirled to dissolve the contents until homogeneity. The HPLC-MS system was controlled with the Agilent MassHunter Workstation software version B.06.00 (Agilent, Santa Clara, CA, USA). For peak integration and quantitative calculation, the Agilent MassHunter Quantitative Analysis software version B.06.00 was used.

### 2.3. Body Composition Analysis

The Tanita Multi-Frequency Body Composition Analyzer MC-180MA (Tanita Corporation, Tokyo, Japan), a weighing instrument that uses bioelectrical impedance analysis to screen body fat and composition, was used to assess body composition. This instrument had been validated against other weighing methods and was repeatedly checked in relation to the reference standards of dual-energy X-ray absorptiometry (DEXA) [19].

Fat mass and fat-free mass were measured. Visceral fat was indirectly estimated and results were given as a specific rating—visceral fat rating (VFR; 0 ± 59; no units). A rate of 1–12 is considered healthy while a rate of 13–59 indicates an excess level of visceral fat. VFR is extensively used in medical research for indirect visceral fat measurement in adults [20].

### 2.4. Ethics

This study was reviewed and approved by the Ethics and Research Committee of Virgen de la Victoria Clinical University Hospital, Málaga, Spain on 12 December 2012 (Ethics Committee Code CMCS240281) and was conducted according to the principles of the Declaration of Helsinki. The participants (who were all volunteers) provided signed consent after being fully informed of the study goal and its characteristics.

### 2.5. Statistical Analysis

Statistical analyses were performed using the IBM SPSS Statistics version 25.0 (IBM Corporation, Armonk, NY, USA). An ANOVA test followed by a Bonferroni post-hoc test was used to compare basal characteristics of the subgroups. We performed a Pearson’s correlation test to evaluate the relationships among the selected variables. Multivariate linear regression analysis was used to examine the associations of demographic, physical, medical, and biochemical factors with 17-OH progesterone levels. Those variables with a *p* value <0.25 in the bivariate model were entered in the multivariate model, and a parsimonious multivariate lineal regression model was constructed, taking into account multicollinearity (through the variance inflation factor). The best model was selected according to the Akaike information criterion, and models with multicollinearity were excluded. Statistical significance was established for *p*-values < 0.05. 

## 3. Results

### 3.1. Study Population

We included 266 non-diabetic adult obese men <50 years in this study. However, according to serum 17-OH progesterone levels, we excluded 13 subjects due to increased circulating levels of 17-OH progesterone above the reference range (>2 ng/mL (6.06 nmol/L)), because these abnormal results cannot completely exclude non-classic congenital adrenal hyperplasia diagnosis, in compliance with literature-established normal ranges according to age and gender [21,22,23]. Thus, the final sample for this study comprised 253 non-diabetic obese men <50 years.

### 3.2. Characteristics of the Study Population

Comparative analysis of clinical characteristics and laboratory parameters of participants according to tertiles of body fat percentage is shown in Table 1. Briefly, in addition to the expected significant increase in BMI, VFR, glucose, HbA1c, hs-CRP, and insulin resistance with increasing body fat percentage, we found that not only TT and FT, but also mean 17-OH progesterone levels, significantly decreased across the body fat percentage continuum. Serum levels of 17-OH progesterone were significantly different between tertiles of body fat percentage (*p* < 0.001). Specifically, mean circulating 17-OH progesterone concentrations were significantly lower in tertile 3 of body fat percentage in comparison with tertile 1 (0.74 ng/mL vs. 0.94 ng/mL, *p* < 0.01; Bonferroni correction) and tertile 2 (0.74 ng/mL vs. 0.89 ng/mL, *p* = 0.02; Bonferroni correction). No differences were found between tertile 1 and tertile 2 of body fat percentage (0.89 ng/mL vs. 0.94 ng/mL, *p* = 0.638). Similar results were found when we evaluated the relationship between the BMI classification and clinical characteristics/laboratory parameters (Supplemental Table S1). In addition, a comparative analysis of 17-OH progesterone levels by tertiles of different measures of adiposity (BMI, WC, and VFR), found decreased 17-OH progesterone levels with the increment of these adiposity markers (Table 2). 

### 3.3. Correlation Analysis between 17-OH Progesterone and Other Variables

A significant negative correlation was detected between serum 17-OH progesterone and weight (*p* < 0.001), body fat (kg and percentage (*p* < 0.001)), BMI (*p* < 0.001), WC (*p* < 0.01), VFR (*p* < 0.001), insulin (*p* < 0.001), and HOMA-IR (*p* < 0.001). A significant positive correlation was found between serum 17-OH progesterone and LH (*p* = 0.001), fat-free mass percentage (*p* = 0.001), and TT and FT (*p* < 0.001) (Table 3). Also, the correlation coefficient between 17-OH progesterone and FT was determined to be 0.450, while the correlation coefficient between 17-OH progesterone and TT was 0.537.

### 3.4. Factors Associated with 17-OH Progesterone Levels

We performed a multiple linear regression analysis to explore factors associated with serum 17-OH progesterone levels (Table 4). The model that best explained serum 17-OH progesterone levels comprised age, body fat percentage, ACTH, HOMA-IR, LH, and FT (F test < 0.01), with an adjusted R^2^ of 0.28. 

Body fat percentage (Beta −0.128, *p* = 0.04) and HOMA-IR (Beta −0.148, *p* = 0.013) was inversely associated with 17-OH progesterone levels, whereas FT levels (Beta 0.382, *p* < 0.001) and ACTH concentrations (Beta 0.125, *p* = 0.032) were positively linked to 17-OH progesterone levels. On the other hand, neither age nor LH levels influenced serum 17-OH progesterone levels (Table 3). 

## 4. Discussion

Our results reveal that circulating 17-OH progesterone levels decrease in parallel with the increase of body fat percentage in non-diabetic young men with obesity. Also, we found that 17-OH progesterone concentrations were independently related to visceral fat, insulin resistance, FT and ACTH levels. 

Obesity, particularly when associated with visceral adiposity accumulation, has a major influence on androgen metabolism, with different implications with respect to sex; obesity in men is associated with a decrease of testosterone, whereas obese women are prone to developing functional hyperandrogenism [24]. While the relationship between low serum testosterone levels and male obesity is firmly established [4,5,25,26] the dynamics of distinct adrenal and gonadal androgens in obesity, specifically 17-OH progesterone, have attracted less interest.

In agreement with the preceding research, our results showed decreased serum 17-OH progesterone concentrations in obese men. Importantly, previous studies presented a reduced sample size and were focused on different circulating hormones in obesity, but included 17-OH progesterone as part of their analyses. Blanchette et al. [14] detected a negative correlation between 17-OH progesterone and BMI in a population of 38 lean to morbidly-obese men between 23 and 61 years old. Isidori et al. [15] investigated leptin impact on several androgens in 28 obese men aged 18–58 years matched with 10 non-obese controls, finding significant differences between both obese men vs. non-obese controls and moderately obese men (BMI 30–40 kg/m^2^) vs. morbidly obese men (>40 kg/m^2^) in 17-OH progesterone levels. In addition, they also found a significant negative correlation between BMI and 17-OH progesterone levels. These results were concordant with those found by Damgaard-Olesen et al., who showed significantly-lower mean serum 17-OH progesterone levels when compared to obese, overweight, and normal-weight subjects [16]. Moreover, similarly to that observed by us, they did not find significant associations with age for 17-OH progesterone. 

**Figure 1:**
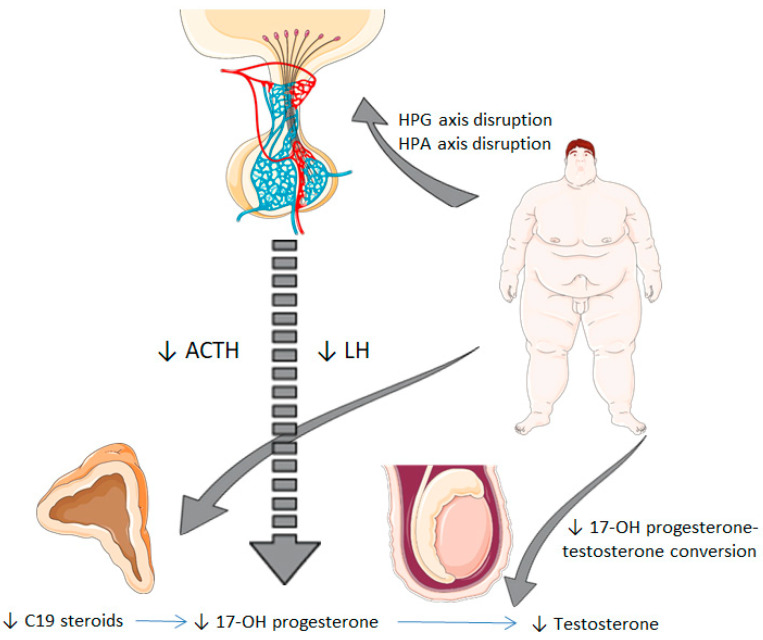
Potential mechanisms from obesity to decreased serum 17-hydroxyprogesterone levels. Obesity is associated with a disruption of the hypothalamic–pituitary–gonadal axis, which involves an impaired synthesis of LH. This fact leads to reduced testicular synthesis of 17-OH progesterone and testosterone. Besides, obesity could also alter the hypothalamic–pituitary–adrenal axis at a central level (impaired secretion of ACTH) and adrenal level (reduced synthesis of C19 steroids), which results in decreased 17-OH progesterone levels. HPG, hypothalamic-pituitary-gonadal; HPA, hypothalamic-pituitary-adrenal; LH: luteinizing hormone; ACTH: adrenocorticotropic hormone; 17-OH progesterone, 17-hydroxyprogesterone.

Low testosterone levels in obesity have been widely reported before, as obesity is the most prevalent cause of hypogonadotropic hypogonadism [3]. Our study population presented progressively-decreased levels of both TT and FT according to the tertiles of body fat percentage. Similarly, serum 17-OH progesterone levels were significantly lower in the tertile 3 of body fat percentage. Besides, 17-OH progesterone levels were shown to be positively associated with circulating TT and FT levels in the correlation analysis, as well as with LH. FT and ACTH were independently-associated factors predicting serum 17-OH progesterone levels in the linear regression analysis. Our findings may suggest that 17-OH progesterone levels follow a similar pattern to testosterone concentrations in obesity and that a connection between the deficiency of these two hormones in obesity could exist. In this line, we hypothesize there are two main mechanisms linking obesity to low serum 17-OH progesterone levels (Figure 1). On the one hand, obesity is associated with a global impairment of androgen synthesis by the Leydig cell as a consequence of alterations in gonadotropin secretion [27]. 17-OH progesterone is predominantly produced by the Leydig cells in testes through LH stimulation, and the adrenal glands are responsible for the remaining biosynthesis [8,9,11]. Therefore hypothalamic–pituitary–gonadal axis disruption in obesity could trigger a diminished secretion of 17-OH progesterone and testosterone. Furthermore, low serum 17-OH progesterone levels could constitute an early step in conditioning decreased testosterone levels in obesity. It has also been postulated that obesity alters 17-20-lyase activity, the enzyme that converts 17-OH progesterone into testosterone, causing an elevated 17-OH progesterone/testosterone ratio with both decreased 17-OH progesterone and testosterone levels with respect to healthy subjects after LH/hCG stimulation [15], a fact that could reinforce the connection between both androgens. 

On the other hand, adrenal synthesis of 17-OH progesterone could be compromised in a similar form. Previous research has demonstrated that there is a decreased C19 steroid production in the adrenal cortex in obese men [13]. A study reported elevated leptin levels in obesity can dysregulate the hypothalamic–pituitary–adrenal axis at adrenal level, causing reduced steroidogenesis and molecular expression of steroid enzymes, including 17 alpha-hydroxylase activity, the enzyme that converts progesterone to 17-OH progesterone [28]. In light of our results, 17-OH progesterone levels could also be influenced by a hypothalamic–pituitary–adrenal axis disturbance at a central level in some obese individuals, in regard to a ACTH-independent association with 17-OH progesterone in the linear regression analysis, which showed a positive link between these two variables. Hence, it must be noted that decreased serum 17-OH progesterone levels in obesity may be explained not only by a gonadal-impaired synthesis, in the same way as the production of several androgens is affected in this condition, but also by the loss of the hypothalamic–pituitary–adrenal axis integrity.

Circulating 17-OH progesterone levels were negatively associated with several parameters related to metabolic syndrome, as BMI, WC, HOMA-IR, fat mass, and VFR. Indeed, body fat percentage and HOMA-IR predicted 17-OH progesterone concentrations in the linear regression model. Thus, it seems plausible that low 17-OH progesterone levels are not only associated with obesity, but also with the abdominal distribution of fat which is characteristic of the metabolic syndrome. Insulin resistance constitutes another important feature of the metabolic syndrome along with abdominal obesity phenotype, and this condition may lead to decreased serum 17-OH progesterone levels. In a similar fashion, testosterone levels in men correlates with insulin sensitivity [29]. It could be feasible to think of a reciprocal influence between the components associated with the metabolic syndrome and 17-OH progesterone too, in the line of the presumable bidirectional association nature described in testosterone-deficiency models [22,23,30,31]. In consequence, 17-OH progesterone could have an impact on body composition and fat distribution, and decreased circulating levels of this hormone in obesity could perpetuate a vicious circle of visceral adiposity accumulation. 

The main limitation of our study was the cross-sectional nature of its design, which prevented us from establishing causality. Therefore, our results can only suggest associations and new hypothesis which should be corroborated in prospective studies. Another limitation of this study is that our results cannot be extrapolated to women. In this line, although obesity has been associated with an increase in androgen levels in obese women, especially testosterone, in a recent study conducted in obese and nonobese women with polycystic ovary syndrome, obese women had lower 17-OH progesterone levels, as compared with lean women [32]. On the other hand, our main strength is the sample size, since as far as we know, this is the largest study assessing circulating 17-OH progesterone levels in obese men, while previous analyses only included a limited number of participants. Besides, we only included young obese participants without diabetes or chronic diseases, in order to reduce confounding factors in our measurements. Another strength of this study is the determination of 17-OH progesterone and testosterone by HPLC-MS, which is considered the gold standard method to determine steroid levels. 

In conclusion, we show that adiposity is associated with notably-decreased serum 17-OH progesterone levels, especially amongst those men with higher body fat percentage. We also report that 17-OH progesterone levels in these subjects are negatively related to body fat percentage and insulin resistance, and positively linked to FT and ACTH levels. Further research is needed to confirm the clinical consequences of these findings.

## Figures and Tables

**Table 1:** Characteristics of the study population according to tertiles of body fat percentage.

	Tertile 1 (*n*-84)	Tertile 2 (*n*-84)	Tertile 3 (*n*-85)	*p* Value
Age (years)	38.4 ± 6.3	36± 8.5	36.2 ± 7.6	0.320
Weight (kg)	103.4 ± 10.2 ^a^	117.7 ± 11.3 ^b^	141.4 ± 17.8 ^c^	<0.001
BMI (kg/m^2^)	33.2 ± 2.8 ^a^	37.3 ± 2.5 ^b^	46.8 ± 5.6 ^c^	<0.001
WC (cm)	111.2 ± 5.7 ^a^	121.5 ± 7.7 ^b^	140.8 ± 12.3 ^c^	<0.001
Fat mass (kg)	29.2 ± 3.7 ^a^	40.1 ± 4.7 ^b^	59.4 ± 11.9 ^c^	<0.001
Fat mass (%)	28.2 ± 2.1 ^a^	34 ± 1.2 ^b^	41.6 ± 3.4 ^c^	<0.001
Fat-free mass (kg)	72.9 ± 5.6 ^a^	77.1 ± 7.1 ^b^	81.8 ± 7.3 ^c^	<0.001
Fat- free mass (%)	70.7 ± 3.7 ^a^	65.5 ± 1.8 ^b^	58.2 ± 3.4 ^c^	<0.001
VFR (points)	12.6 ± 2 ^a^	16.7 ± 2.5 ^b^	26.1 ± 5.4 ^c^	<0.001
Glucose (mg/dL)	90.7 ± 8.2 ^a^	92.6 ± 11.2 ^a,b^	94.5 ± 9.8 ^b^	0.048
HbA1c (%)	5.3 ± 0.3 ^a^	5.4 ± 0.4 ^a^	5.5 ± 0.4 ^b^	0.035
Triglycerides (mg/dL)	150.7 ± 81.1	149 ± 75.8	157.5 ± 83.5	0.773
HDL-c (mg/dL)	42.3 ± 7.6	42.2 ± 10.4	41.1 ± 9.5	0.676
LDL-c (mg/dL)	118 ± 28	112 ± 31.4	110.5 ± 29.4	0.229
hs-CRP (mg/dL)	1.6 ± 1.9 ^a^	2.9 ± 4.5 ^a^	5.7 ± 8.5 ^b^	<0.001
Insulin (uIU/mL)	13.5 ± 6.4 ^a^	20 ± 13.2 ^b^	27.1 ± 21.5 ^c^	<0.001
HOMA-IR	3.1 ± 1.6 ^a^	4.8 ± 3.9 ^b^	6.5 ± 6 ^c^	<0.001
ACTH (pg/mL)	25.2 ± 15.9	27 ± 14.2	29.7 ± 15.1	0.172
LH (mUI /mL)	3.8 ± 1.7	4 ± 2.7	3.5 ± 1.7	0.371
TT (ng/mL)	4.1± 1.4 ^a^	3.8 ± 1.2 ^a^	3.2 ± 1.2 ^b^	<0.001
FT (pg/mL)	94.6 ± 28.3 ^a^	92.3 ± 27.7 ^a^	77.7 ± 24.5 ^b^	<0.001
17-OH progesterone (ng/mL)	0.94 ± 0.4 ^a^	0.89 ± 0.33 ^a^	0.74 ± 0.31 ^b^	<0.001

Values are presented as mean ± SD (standard deviation). *p* values were calculated for differences between groups using ANOVA test, considering *p* < 0.05 significant. Means denoted by a different letter indicate significant differences between groups (*p* < 0.05). BMI, body mass index; WC, waist circumference; VFR, visceral fat rating; HbA1c, hemoglobin A1c; HDL-c, high-density lipoprotein cholesterol; LDL-c, low-density lipoprotein cholesterol; hs-CRP, high-sensitivity C-reactive protein; HOMA-IR, homeostatic model assessment of insulin resistance; ACTH, adrenocorticotrophic hormone; LH, luteinizing hormone; TT, total testosterone; FT, free testosterone. Reference intervals: ACTH, 5–50 pg/mL; LH, 1.5–7.7 mUI /mL; TT, ≥3.5 ng/mL; FT, ≥70 pg/mL; 17-OH progesterone, ≤2 ng/mL.

**Table 2:** 17-Hydroxyprogesterone levels (ng/mL) by tertiles of different measures of adiposity.

	Tertile 1	Tertile 2	Tertile 3	*p* Value
BMI	0.94 ± 0.38 ^a^	0.88 ± 0.33 ^a^	0.73 ± 0.36 ^b^	<0.001
WC	0.94 ± 0.36 ^a^	0.89 ± 0.35 ^a^	0.71 ± 0.31 ^b^	<0.001
VFR	0.93 ± 0.39 ^a^	0.91 ± 0.34 ^a^	0.70 ± 0.36 ^b^	<0.001

Values of 17-hydroxyprogesterone are presented as mean ± SD SD (standard deviation). *p* values were calculated for differences between groups using ANOVA test, considering *p* < 0.05 significant. Means denoted by a different letter indicate significant differences between groups (*p* < 0.05). BMI, body mass index; WC, waist circumference; VFR, visceral fat rating.

**Table 3:** Correlation coefficients among 17-OH progesterone and anthropometric, biochemical, and hormonal parameters.

	17-OH Progesterone	
	r	*p* Value
Age (years)	−0.120	0.058
Weight (kg)	−0.245	<0.001
BMI (kg/m^2^)	−0.299	<0.001
WC (cm)	−0.297	<0.001
VFR (points)	−0.322	<0.001
Fat mass (kg)	−0.284	<0.001
Fat mass (%)	−0.290	<0.001
Fat-free mass (%)	0.270	<0.001
Glucose (mg/dL)	−0.086	0.171
Insulin (uIU/mL)	−0.256	<0.001
HOMA-IR	−0.243	<0.001
hs-CRP (mg/dL)	−0.095	0.137
ACTH (pg/mL)	0.077	0.232
LH (mUI/mL)	0.214	0.001
TT (mg/dL)	0.537	<0.001
FT (pg/mL)	0.450	<0.001

Bold values mean significant statistical association in Pearson’s correlation test. BMI, body mass index; WC, waist circumference; VFR, visceral fat rating; HOMA-IR, homeostatic model assessment of insulin resistance; hs-CRP, high-sensitivity C-reactive protein; ACTH, adrenocorticotrophic hormone; LH, luteinizing hormone; FT, free testosterone; TT, total testosterone.

**Table 4:** Multiple linear regression analysis: factors related to 17-OH progesterone levels.

	B (SE)	Beta	T Statistic	*p* Value
Age (years)	−0.001 (0.003)	−0.031	−0.536	0.592
Body fat (%)	−0.008 (0.004)	−0.128	−2.029	0.044
ACTH (pg/mL)	0.003 (0.001)	0.125	2.158	0.032
HOMA-IR	−0.012 (0.005)	−0.148	−2.514	0.013
LH (mUI/mL)	0.017 (0.010)	0.102	1.771	0.078
FT (pg/mL)	0.005 (0.001)	0.382	6.020	<0.001

Unstandardized coefficients (B) of the linear regression model with SE, standardized coefficients (Beta), T Statistic, and *p* values. ACTH, adrenocorticotrophic hormone; HOMA-IR, homeostatic model assessment of insulin resistance; LH, luteinizing hormone; FT, free testosterone.

## Supplementary Materials

The following supplemental material is available online: https://www.mdpi.com/2077-0383/9/12/3873/s1, Supplemental Table S1: Characteristics of the study population according to body mass index (BMI) classification.

## Abbreviations

ACTH	Adrenocorticotropic hormone
BMI	Body mass index
FT	Free testosterone
HOMA-IR	Homeostatic model assessment of insulin resistance
HPLC-MS	High-performance liquid chromatography mass spectrometry
hS-CRP	High-sensitivity C-reactive protein
LH	Luteinizing hormone
17-OHprogesterone	17-hydroxyprogesterone
TT	Total testosterone
VFR	Visceral fat rating
WC	Waist circumference
