# Supplementary material for: Adiposity is Associated with Decreased Serum 17-Hydroxyprogesterone Levels in Non-Diabetic Obese Men Aged 18–49: A Cross-Sectional Study
Source: J Clin Med. 2020 Nov 28;9(12):3873. doi: 10.3390/jcm9123873 (PMC7760398; doi:10.3390/jcm9123873)
Supplement: Supplementary file 1 [file jcm-09-03873-s001.pdf]

Supplemental Table S1. Characteristics of the study population according to body mass index (BMI) classification

	BMI 30-34.9 kg/m^2 (n = 81)	BMI 35-39.9 kg/m^2 (n = 80)	BMI ≥40 kg/m^2 (n = 92)	p value
Age (years)	38 ± 6.5	36.2± 8.2	36.9 ± 8	0.320
Weight (kg)	101.3 ± 8.4^a	118.1 ± 9.8^b	140.6 ± 16.8^c	**<0.001**
BMI (kg/m^2)	32.4 ± 1.2^a	37.5 ± 1.5^b	46.5 ± 5.3^c	**<0.001**
WC (cm)	110.5 ± 5.1^a	121.8 ± 7.5^b	139.2 ± 12.9^c	**<0.001**
Fat mass (kg)	29.2 ± 4.2^a	39.8 ± 4.8^b	58 ± 12.6^c	**<0.001**
Fat mass (%)	28.7 ± 2.7^a	33.7 ± 2.4^b	40.8 ± 4.6^c	**<0.001**
Fat-free mass (kg)	71.9 ± 5.8^a	77.4 ± 6.5^b	82 ± 6.9^c	**<0.001**
Fat- free mass (%)	70.9 ± 2.7^a	65.7 ± 2.3^b	58.4 ± 3.7^c	**<0.001**
VFR (points)	12.6 ± 2.0^a	16.6 ± 2.1^b	25.5 ± 5.7^c	**<0.001**
Glucose (mg/dl)	90.6 ± 7.9^a	91.9 ± 10.9^{a,b}	95 ± 10.3^b	**0.009**
HbA1c (%)	5.3 ± 0.3^a	5.4 ± 0.3^a	5.5 ± 0.4^b	**<0.001**
Triglycerides (mg/dl)	154.2 ± 81.7	143 ± 73.1	162.3 ± 83.8	0.291
HDL-c (mg/dl)	42.1 ± 7.6	42.2 ± 9.1	41.9 ± 9.2	0.906
LDL-c (mg/dl)	119 ± 26.5	111 ± 31.2	113.8 ± 29.5	0.160
hs-CRP (mg/dl)	2.1 ± 4.1^a	2.4 ± 2.9^a	5.2 ± 8^b	**<0.001**
Insulin (uIU/ml)	14.4 ± 7.4^a	19 ± 12.9^a	26.3 ± 20.6^b	**<0.001**
HOMA-IR	3.3 ± 1.8^a	4.5 ± 3.8^a	6.3 ± 5.7^b	**<0.001**
ACTH (pg/ml)	25.6 ± 16.2	26.8 ± 14.3	28.6 ± 14.9	0.430
LH (mUI /ml)	4.1 ± 1.2	3.8 ± 1.2	3.3 ± 1.4	0.085
TT (ng/ml)	4.1± 1.2^a	3.9 ± 1.4^a	3.3 ± 1.3^b	**<0.001**
FT (pg/ml)	96.4 ± 27.1^a	92.2 ± 29.4^a	79.4 ± 25.6^b	**<0.001**
17-OH progesterone (ng/ml)	0.95 ± 0.38^a	0.88 ± 0.35^a	0.74 ± 0.30^b	**<0.001**

Values are presented as mean ± SD (standard deviation). p Values were calculated for differences between groups using ANOVA test, considering $p < 0.05$ significant. Means denoted by a different letter indicate significant differences between groups ($p < 0.05$). BMI, body mass index; WC, waist circumference; VFR, visceral fat rating; HbA1c, haemoglobin A1c; HDL-c, high-density lipoprotein cholesterol; LDL-c, low-density lipoprotein cholesterol; hs-CRP, high-sensitivity C-reactive protein; HOMA-IR, homeostatic model assessment of insulin resistance; ACTH, adrenocorticotrophic hormone; LH, luteinizing hormone; TT, total testosterone; FT, free testosterone. Reference intervals: ACTH, 5-50 pg/ml; LH, 1.5- 7.7 mUI /ml; TT, ≥3.5 ng/ml; FT, ≥70 pg/ml; 17-OH progesterone, ≤ 2 ng/ml.
